# Comparison of dynamic mode decomposition with other data-driven models for lung cancer incidence rate prediction

**DOI:** 10.3389/fpubh.2025.1472398

**Published:** 2025-04-25

**Authors:** L. Raymond Guo, Jifu Tan, M. Courtney Hughes

**Affiliations:** ^1^Department of Interdisciplinary Sciences, Northern Illinois University, DeKalb, IL, United States; ^2^Department of Mechanical Engineering, Northern Illinois University, DeKalb, IL, United States; ^3^Department of Public Health, Northern Illinois University, DeKalb, IL, United States

**Keywords:** machine learning, dynamic mode decomposition, lung cancer, public health data, random forest, gradient boosting machine

## Abstract

**Introduction:**

Public health data analysis is critical to understanding disease trends. Existing analysis methods struggle with the complexity of public health data, which includes both location and time factors. Machine learning offers powerful tools but can be computationally expensive and require specialized knowledge. Dynamic mode decomposition (DMD) is an alternative that offers efficient analysis with fewer resources. This study explores applying DMD in public health using lung cancer data and compares it with other machine learning models.

**Methods:**

We analyzed lung cancer incidence data (2000–2021) from 1,013 US counties. Machine learning models (random forest, gradient boosting machine, support vector machine) were trained and optimized on the training data. We also employed time series, a linear regression model, and DMD for comparison. All models were evaluated based on their ability to predict 2021 lung cancer incidence rates.

**Results:**

The time series model achieved the lowest root mean squared error, followed by random forest. Meanwhile, DMD had an RMSE similar to that of Random Forest. Nearly all counties in Kentucky had higher lung cancer incidence rates, while states like California, New Mexico, Utah, and Idaho showed lower trends.

**Conclusion:**

In summary, DMD offers a promising alternative for public health professionals to capture underlying trends and potentially have lower computational demands compared to other machine learning models.

## 1 Introduction

Public health data is multidimensional, encompassing factors like geolocation and time. Spatial–temporal analysis of public health data helps us understand disease patterns, identify vulnerable populations, and design interventions. Time series models were traditionally used for temporal data, but they lack insights into geospatial patterns. Machine learning (ML) uses algorithms trained on data sets to create models that enable machines to perform tasks that would otherwise only be possible for humans. ML empowers computers to learn from data, identify patterns, predict outcomes, and discover the hidden coherent structures among complex data. ML algorithms like random forest (RF) and gradient boosting machine (GBM) excel at finding patterns in complex data, revealing connections between demographics, movement, and disease spread. This allows for more precise outbreak predictions and targeted interventions. Furthermore, support vector machines (SVMs) effectively classify data by drawing a line (or hyperplane) that best divides the data into two groups, maximizing the distance between the groups. SVMs are valuable even with limited samples for disease-type classification of individual risk prediction.

While powerful for prediction and temporal analysis, the existing models (RF, GBM, SVM) often require additional geospatial analysis. Their effective use requires specialized data science and machine learning expertise. Additionally, training complex models demands significant computational resources, which may not be universally accessible, especially for public health practitioners in resource-limited settings, hindering early detection and response times. Furthermore, the interpretability of ML models is limited. In contrast to ML, dynamic mode decomposition (DMD) is a data-driven approach for analyzing dynamic systems by obtaining coherent spatial–temporal modes to efficiently analyze the data from complex systems with clear perspectives. It decomposes complex data into simpler modes, revealing the underlying processes without requiring a traditional physical model. While ML excels at prediction, DMD provides a deeper understanding of the data’s dynamic patterns. DMD was originally developed in the fluid mechanics community to discover low dimensional models of coherent structures (1). Later, it was applied to analyze other data such as power grid (2), influenza and measles (3), and COVID-19 (4). DMD has the advantages of being low-cost and using minimal calculating power while having the capacity to explore underlying data patterns. It reduces the complexity of high-dimensional data by decomposing it into a set of dynamic modes, each associated with a specific oscillation frequency and decay/growth rate that enables revealing underlying patterns responsible for observed behavior. DMD allows researchers to analyze the data with less computational power, saving time. It also offers a compelling combination of low-dimensional DMD interpretable information, allowing researchers to gain insights into the system’s dynamics. This approach is particularly valuable compared to traditional machine learning models that often require extensive computational resources and are less explainable. DMD’s computational efficiency and relative ease of use make it a potentially powerful tool for public health practitioners with interpretable modes. This opens doors to applying data-driven insights in public health settings with reduced barriers to entry.

Lung cancer is the second most common cancer in the United States, and the National Cancer Institute predicts 234,580 new lung cancer cases in 2024 (5). The annual per-patient cost of medical services for patients with lung cancer ranges from $12,200 to $118,000 annually (6), with the greatest financial burden occurring at the time of initial diagnosis and the last year of life. This poses a significant burden to patients, caregivers, and healthcare systems. Previous studies used machine learning models to predict lung cancer incidence rates, often including cancer-associated predictors and determinants. In practice, collecting and validating such lung cancer data can be time consuming.

No single ML model is superior for cancer rate prediction, with different researchers determining different ML models to be superior in different studies (7). RF (7, 8) and neural networks (7, 9) are common models for predicting lung cancer incidence rates. RF, while powerful, can be a “black box” for public health professionals unfamiliar with advanced statistics, making it difficult to interpret how the model arrives at its conclusions. On the other hand, neural network models require multiple predictors, especially at a larger scale, to achieve efficient predictions. With neural networks, it can also be challenging to collect high-dimensional data quickly across different geographic locations. Other models, such as SVM (8) and GBM (10) have also been used to predict lung cancer with highly accurate predictions. However, their implementation can require specialized software and parameter tuning, posing a challenge for public health professionals without a strong data science background. There is a need to find a quick and less complex method to predict the temporal trends of lung cancer. This study aimed to apply and test DMD to analyze a large-scale lung cancer incidence dataset at the county level in public health settings to identify hidden temporal patterns, dependencies, and dynamic relationships and assess the prediction ability of DMD compared to other traditional and machine learning-based data analysis methods. We hypothesized that DMD would exhibit comparable prediction accuracy to RF and SVM.

## 2 Methods

### 2.1 Data source

We collected county-level lung cancer age-adjusted incidence rates from 2000 to 2021 from 22 state registries within the Surveillance, Epidemiology, and End Results Program (SEER) (11) using SEER*Stat software. We used the Agency for Healthcare Research and Quality (AHRQ) Digital Healthcare Research Checklist to extract the data (12). We obtained the delayed-incidence rate, which has been adjusted for reporting delay. Reporting delay refers to the time between cancer diagnosis and reporting to cancer registries. Analyzing delayed rates can help determine cancer incidence rates and trends more precisely. After removing 15 state-level registries and 55 counties with missing data, we analyzed data from 1,013 counties using TS, RF, GBM, and SVM. We then conducted DMD to compare the results.

### 2.2 Training and testing datasets

We first divided the data into two datasets: training (2000–2020) and testing (2021). We used data from 2000–2019 to build RF, GBM, SVM, and TS models, using the RMSE (root mean squared error) as the measure to optimize the parameters and obtain the lowest RMSE. Then, we applied the trained model to the 2000–2020 data to predict 2021 data and calculated the RMSE for each method.

### 2.3 Random forest and gradient boosting machine

RFs were constructed by fitting multiple decision trees to random subsets of the training data, with each tree using a random selection of features at each split (13). We determined the optimal number of trees and maximum tree depth through a grid search, evaluating models based on Root Mean Squared Error (RMSE) on the training data. GBMs were similarly fit using a grid search to optimize the number of trees and interaction depth, which controls the complexity of allowed interactions between features in the model (2). For both RF and GBM, the final model was trained on the entire training data set using the hyperparameters identified through the grid search. We evaluated the performance of the final models by predicting the target variable (2021) and calculating the RMSE.

### 2.4 Support vector machine (SVM)

We conducted a grid search to tune the hyperparameters of the SVM model. The hyperparameters evaluated were cost and gamma. Cost controls the trade-off between maximizing the margin between the decision boundary and the support vectors and minimizing the training error. Gamma controls the influence of training data points on the decision boundary. We used a radial basis function kernel for the SVM, as it is a common choice for non-linear relationships between features.

The grid search evaluated different combinations of cost values (0.1, 1, 10, 100) and gamma values (0.1, 1, 10, 100). We calculated the root RMSE on the training data to evaluate each model’s performance. We selected the model with the lowest RMSE as the optimal model. The final SVM model was trained on the entire training data set using the hyperparameters identified through the grid search. The final model was then used to predict the 2021 lung cancer incidence rate using 2000–2020 data.

### 2.5 Time series

We applied the exponential smoothing with trend (ETS) model, a popular technique well-suited for capturing trends in time series data. ETS offered a suitable balance between simplicity and effectiveness for this specific analysis focused on capturing trends in incidence rates, especially for public health data analysis to practitioners unfamiliar with complex time series models.

We implemented the ETS model using the “ets” function from the “forecast” R package. The alpha parameter, which controls the weight given to recent observations in the smoothing process, was set to 0.2, which was chosen based on the prior optimization process. For each county, the ETS model was fit to the historical incidence rate data. The resulting model was then used to generate a one-step forecast for the incidence rate in the year 2021.

### 2.6 Linear regression model

We conducted a linear regression model using 2000–2019 data as input and 2020 as the outcome to train the model first. Once we obtained the coefficients, we used 2000–2020 data to predict 2021 age-adjusted mortality rates and calculated the RMSE. We conducted this analysis in R, version 4.3.3.

### 2.7 Dynamic mode decomposition

We organized the incidence rate data into matrix $X=\left[x_{1}\;x_{2}\ldots x_{m}\;\right]$ where $x_{k}$ is the incidence rate at the kth year. Then, we reorganized these data into two matrices, $X_{1}=\left[x_{1}\;x_{2}\ldots x_{m-1}\;\right]$ , $X_{2}=\left[x_{2}\;x_{3}\ldots x_{m}\;\right]$ , where m is the total number of snapshots. Assume $X_{2}$ can be linearly approximated with $X_{1}$ such that $X_{2}\approx AX_{1}$ , then the matrix could then be calculated as $A=X_{2}X_{1}^{†}$ , where $X_{1}^{†}$ is the Moore-Penrose pseudoinverse of $X_{1}$ . We started with the singular value decomposition (SVD) of $X_{1}$


$$X_{1}\approx U\Sigma V^{*}$$

where ∗ denotes the conjugate transpose, $U\in C^{n\times r},\Sigma \in C^{r\times r}$ and is diagonal, $V\in C^{m\times r}$ , n is the total number of the elements in one snapshot, and r is the rank of the reduced SVD approximation to $X_{1}$ . The full matrix A can be computed as $A=X_{2}V\Sigma^{-1}U^{*}$ . In practice, researchers are usually only interested in the leading r eigenvalues and eigenvectors of A , so an order-reduced approximation $\tilde{A}$ can be calculated by projecting the full matrix onto the proper orthogonal decomposition modes, $\tilde{A}=U^{*}\mathrm{AU}=U^{*}X_{2}V\Sigma^{-1}$ . The reduced order matrix defines a linear model ${\tilde{x}}_{k+1}=\tilde{A}{\tilde{x}}_{k}$ where the original vector $x_{k}=U{\tilde{x}}_{k}$ . The spectral decomposition of $\tilde{A}$ can be calculated as $\tilde{A}W=W\Lambda$ , where W are eigenvectors of $\tilde{A}$ , Λ is a diagonal matrix contains eigenvalues $\lambda_{k}$ of the DMD, which are also the eigenvalues of the original matrix A . Finally, the DMD modes for matrix A can be constructed as $\Phi =X_{2}V\Sigma^{-1}W$ . The prediction by DMD for $x_{k}=\Sigma_{j=1}^{r}\phi_{j}\lambda_{j}^{k-1}b_{j}=\Phi \Lambda^{k-1}b$ where $\phi_{j}$ and $\lambda_{j}$ are DMD modes and eigenvalues (or eigenvectors and eigenvalues of the matrix A ); $b_{j}$ is the mode amplitude (1, 14).

Twenty-one years of smoking data were available for the prediction. We used the first 20 years of data (m = 20) to construct the DMD analysis and the last year of data to compare with the prediction by DMD. We followed the Strengthening the Reporting of Observational Studies in Epidemiology (STROBE) reporting guidelines (15).

## 3 Results

### 3.1 2021 county-level lung cancer incidence rate prediction

Our evaluation of different machine learning models for predicting the 2021 lung cancer incidence rate showed TS has the lowest RMSE at 15.3 (Table 1), while RF was 20.5. SVM and GBM performed similarly, with RMSEs being 24.4 and 24.3, respectively. Linear regression had the highest RMSE of 24.7. In general, the predicted rates were higher than the other models. From the box plot, the range and quartile of the predicted values were similar to the actual incidence rate. DMD’s MSE of 20.5 is the same as that of the random forest model. Conversely, the DMD had the highest Spearman correlation coefficient (0.706) among the machine learning models between the predicted and actual values of the 2021 lung cancer incidence rate, while GBM had the lowest (0.589).

**Table 1:** Root mean squared error or predicted 2021 lung cancer incidence rate.

Studied models	RMSE*	Spearman correlation coefficient
Dynamic mode decomposition	20.5	0.706**
Time series	15.3	0.822**
Linear regression	24.7	0.644**
Random forest	20.5	0.654**
Gradient boost machine	24.3	0.589**
Support vector machine	23.4	0.610**

*RMSE: root mean square error. **p < 0.001.

### 3.2 Dynamic mode decomposition

**Figure 1:**
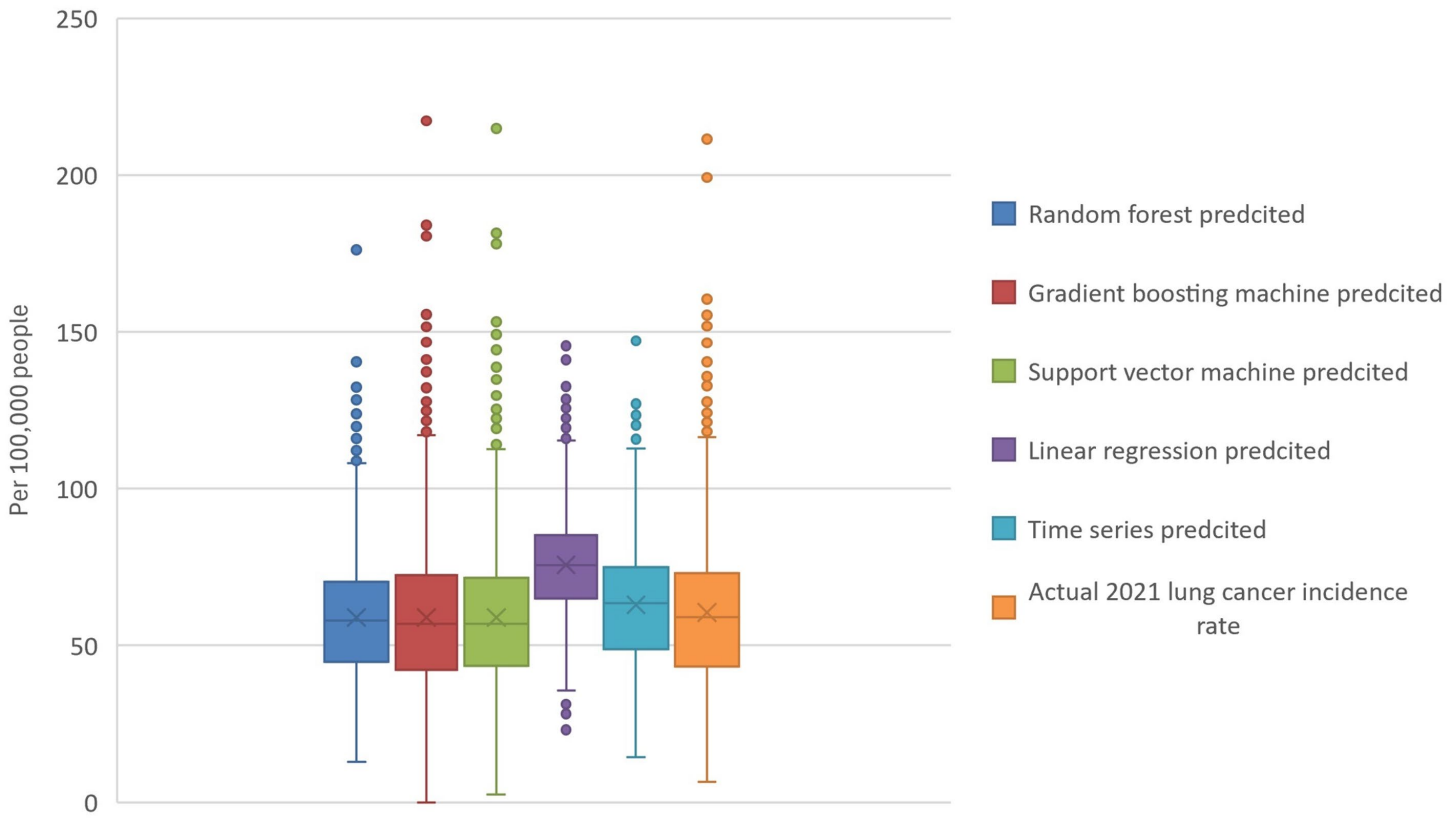
Actual and model-predicted 2021 lung cancer incidence rates. The boxplot visualizes the actual and predicted 2021 lung cancer incidence rates and reveals that random forest, gradient boosting, and support vector machines all have predicted means similar to the actual data. In contrast, linear regression and time series models predict higher values. Additionally, the random forest model exhibits the tightest range of predicted values.

**Figure 2:**
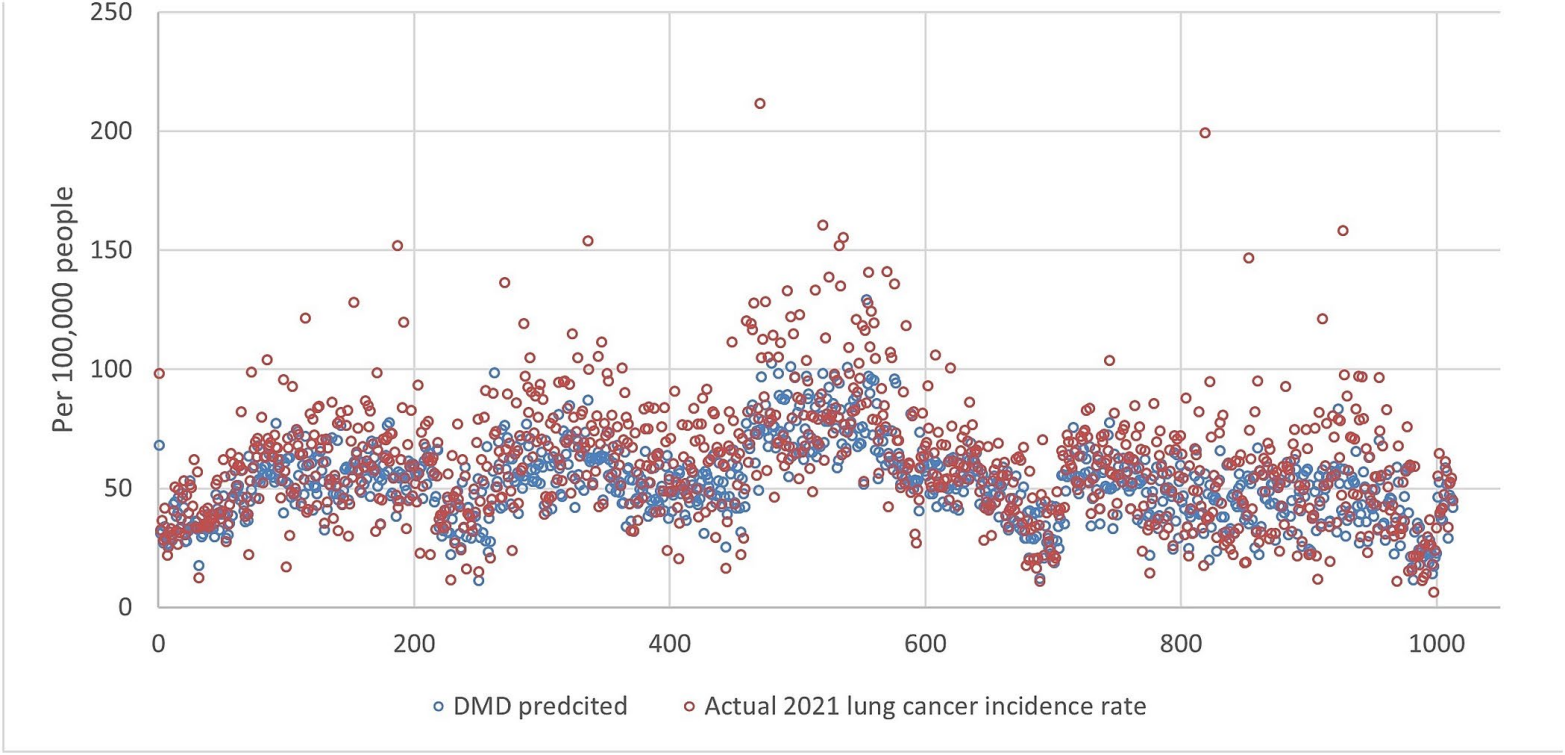
The lung incidence rate for 2021 is compared with dynamic mode decomposition prediction using all ranks. The x-y plot visualizes the DMD predicted (blue dot) versus the actual (red dot) 2021 lung cancer incidence rate. It indicates that the DMD model predicts overall lower values, but the fluctuation trends align with the actual rates.

An example of the prediction using all ranks is shown in Figures 1, 2, indicating that DMD can capture the overall trend of the lung incidence rate in different counties but with under-predictions for high incidence rates.

**Figure 3:**
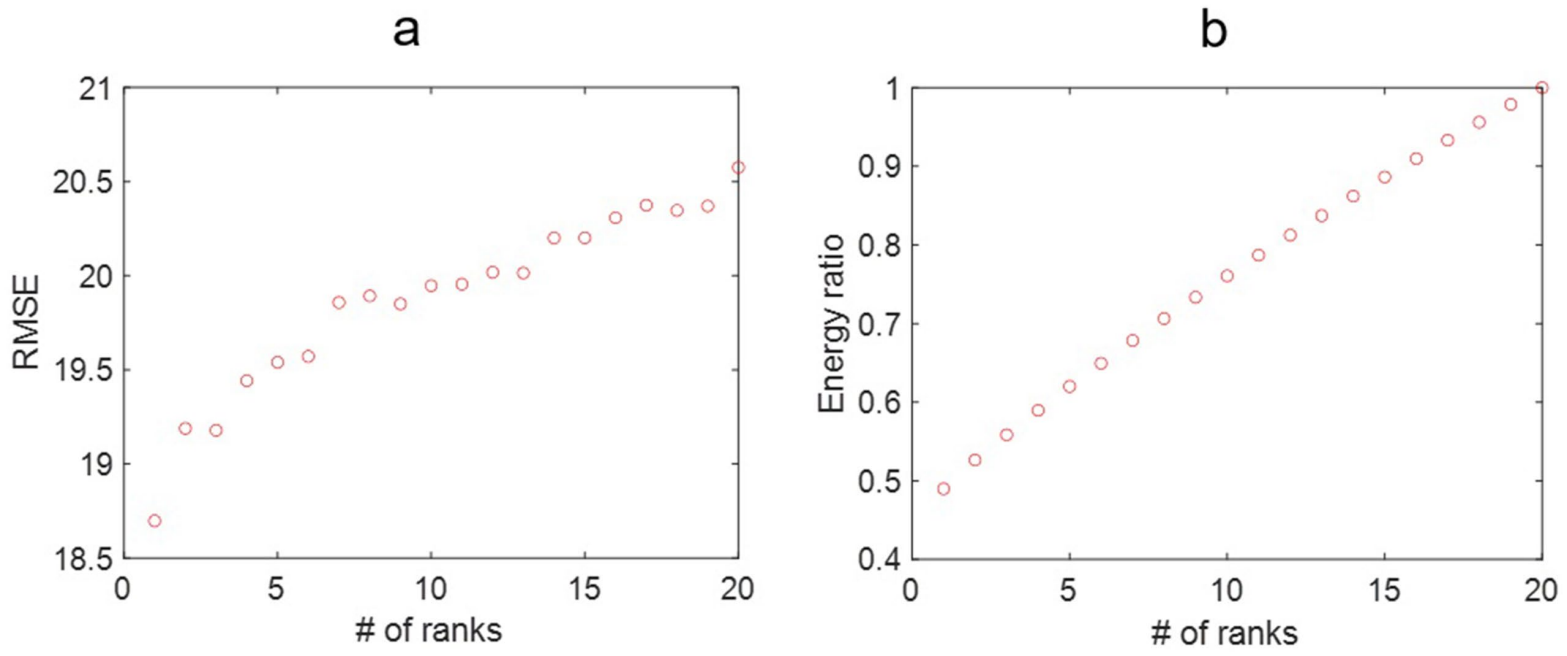
**(a)** The root mean square error (RMSE) for the lung incidence rate for dynamic mode decomposition prediction with different ranks of the mapping matrix. That the RMSE increases with the number of ranks may be associated with the fluctuating nature of the lung incidence rates. **(b)** The energy ratio of the truncated singular value decomposition at different ranks over the original data. More energy is retained in the reconstructed model with increased ranks.

**Figure 4:**
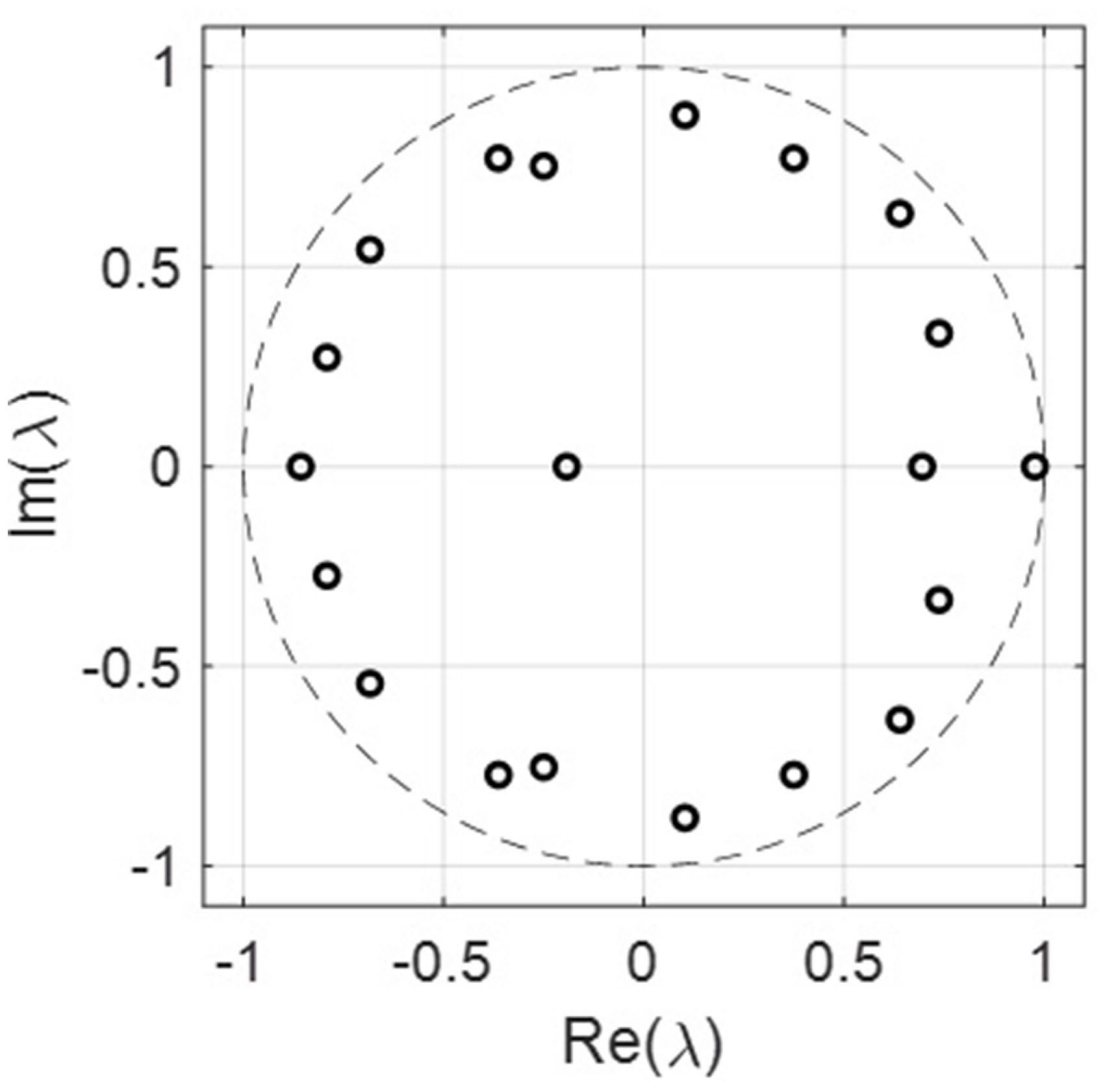
The real and imaginary parts of the eigenvalues for all the 20 ranks. Most of the eigenvalues are within the unit circle, indicating the decaying rate for lung incidence.

We checked the RMSE for the year 2021 with the number of ranks used in SVD, as shown in Figure 3a. In general, the RMSE increased with the ranks, which is surprising as the RMSE usually decreases with the number of ranks used in the reconstruction. This indicates that the incidence rate data may contain noise, and the sample size for 20 years may not be enough to form a linear analysis. Figure 3b shows that the more ranks used during the SVD truncation, the more energy is retained in decomposition. The distribution of eigenvalues shows the dynamic features of the associated modes such as growing, decaying, or oscillating behavior for each mode. The eigenvalues are plotted in Figure 4, where most of the eigenvalues are within the unit circle, indicating the decaying of lung incidence rates over the years. It is consistent with the raw data that the incidence rates are decreasing over the years.

### 3.3 Machine learning and other models

**Figure 5:**
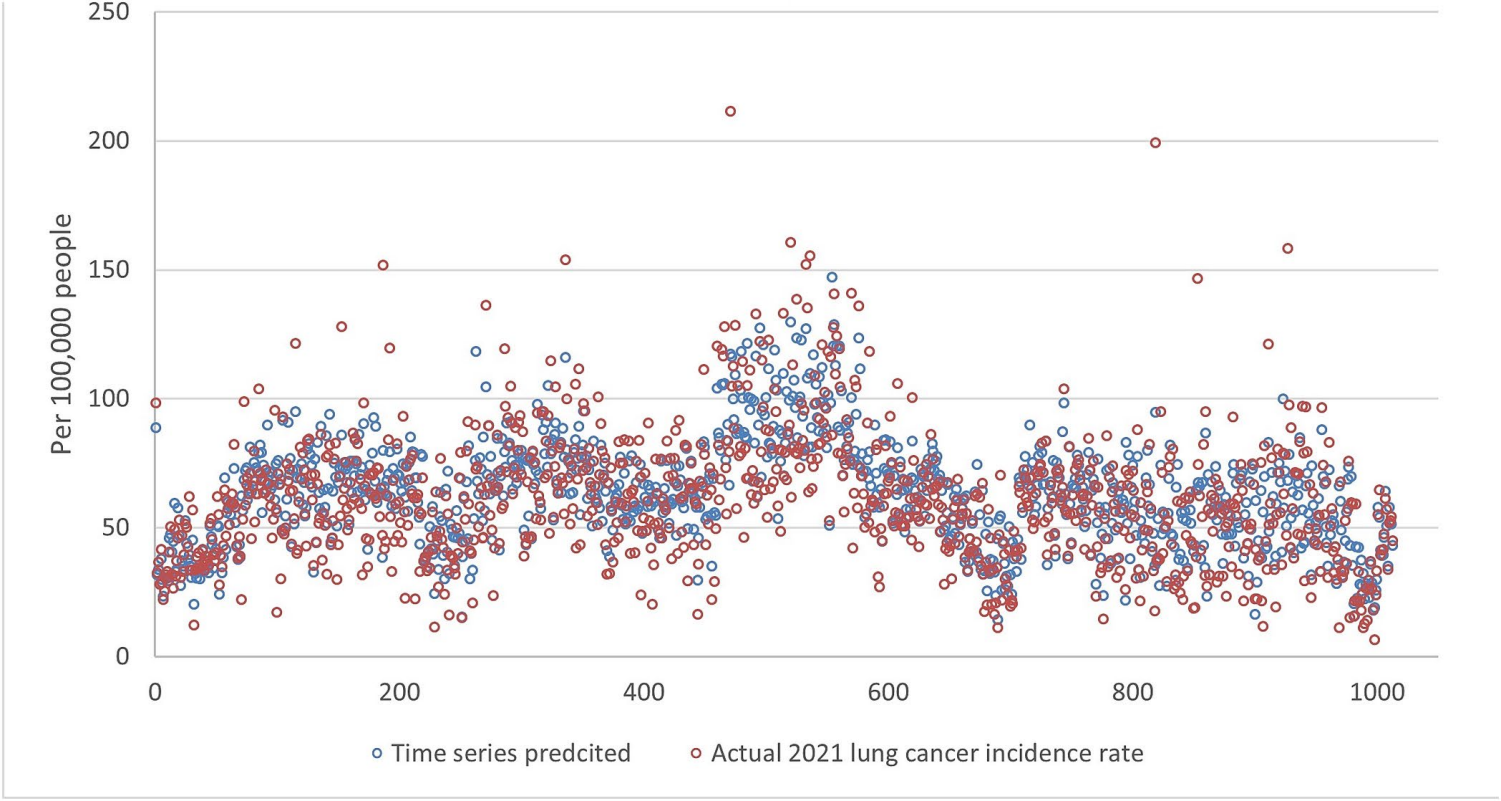
The comparison of the 2021 lung incidence rate with time series prediction using all ranks. The x-y plot visualizes the time series predicted (blue dot) versus the actual (red dot) 2021 lung cancer incidence rate. It indicates random forest predicted values exhibit low variance compared to the actual data. This indicates generally more conservative estimates with less spread.

**Figure 6:**
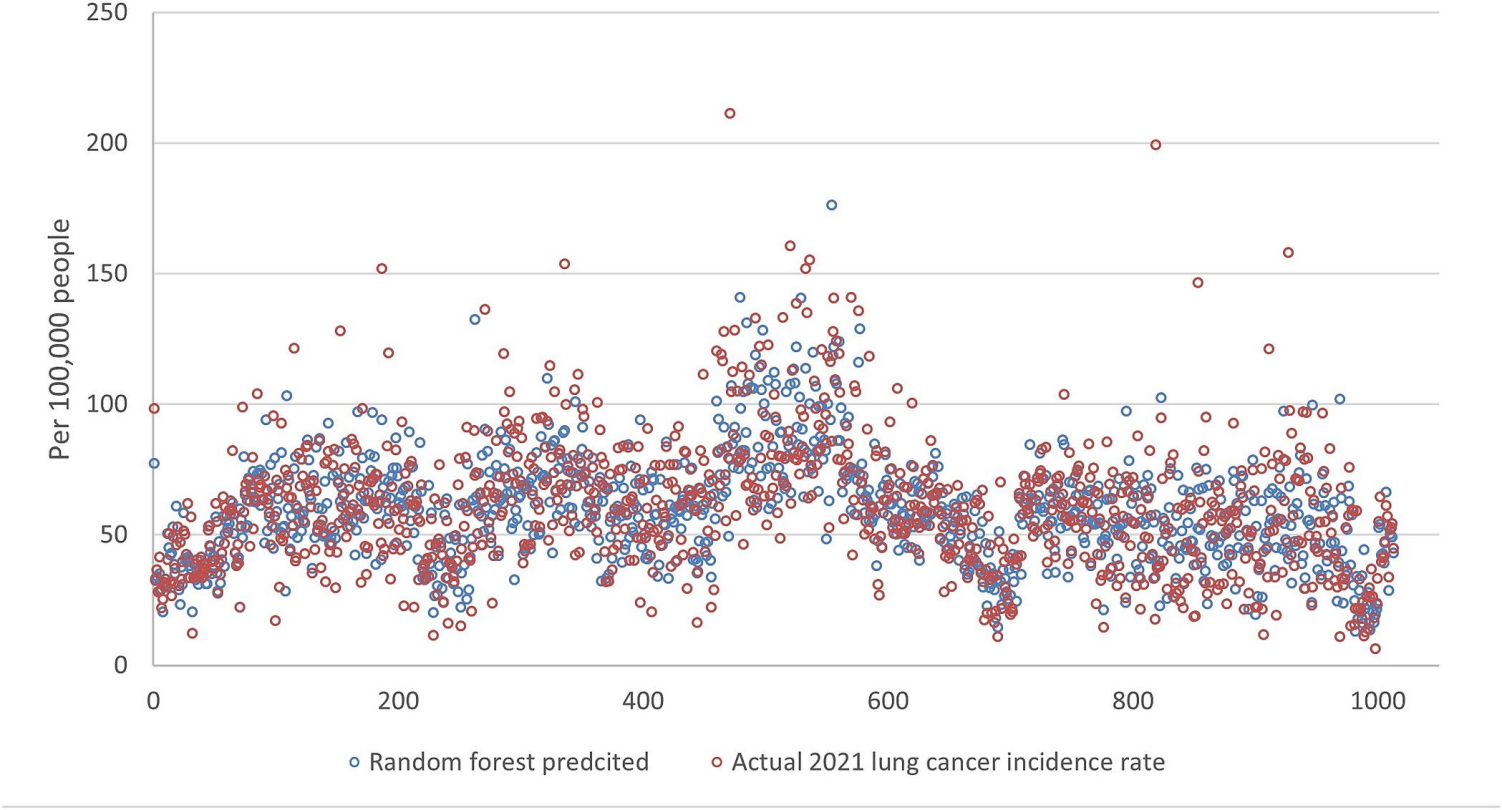
The comparison of the 2021 lung incidence rate with random forest prediction using all ranks. The x-y plot visualizes the random forest predicted (blue dot) versus the actual (red dot) 2021 lung cancer incidence rate. It indicates random forest predicted values have acceptable variance while still matching the overall trend.

**Figure 7:**
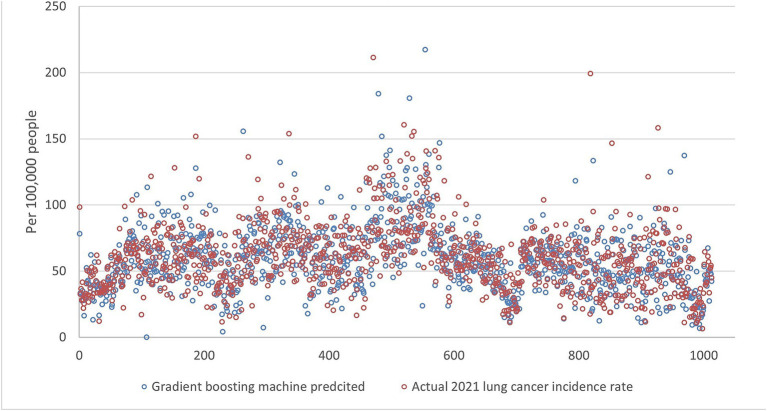
The comparison of the 2021 lung incidence rate with gradient boosting machine prediction using all ranks. The x-y plot visualizes the gradient boosting machine predicted (blue dot) versus the actual (red dot) 2021 lung cancer incidence rate. It indicates gradient boosting machine predicts values with a higher variance, although it captures the overall trend of the real data.

**Figure 8:**
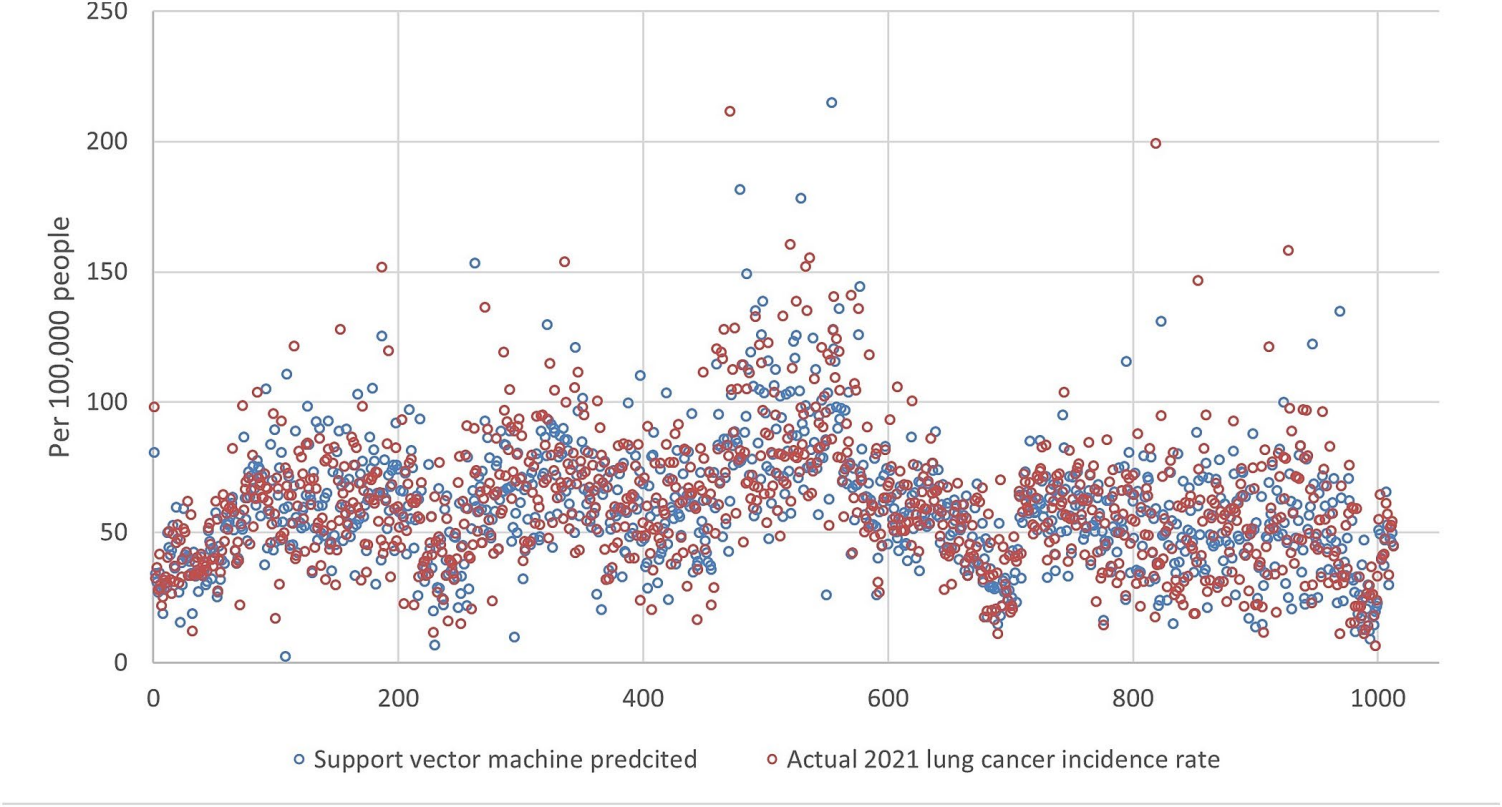
The 2021 lung incidence rate was compared with support vector machine prediction using all ranks. The x-y plot visualizes the support vector machine predicted (blue dot) versus the actual (red dot) 2021 lung cancer incidence rate. It indicates that support vector machine predicted data have high variance and contain more extreme values.

**Figure 9:**
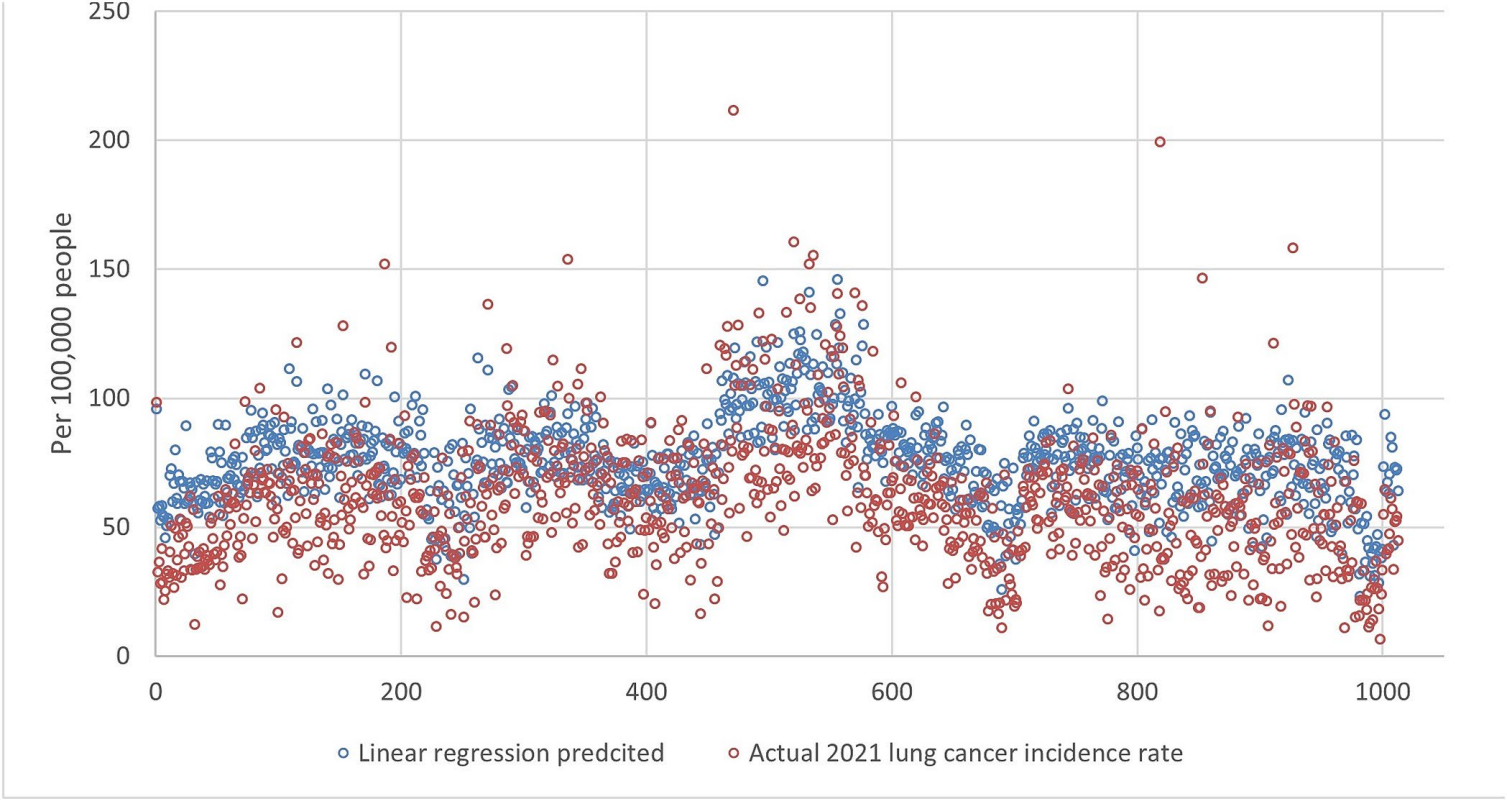
The comparison of the 2021 lung incidence rate with linear regression prediction using all ranks. The x-y plot visualizes the linear regression predicted (blue dot) versus the actual (red dot) 2021 lung cancer incidence rate. It indicates linear regression predicts values with similar fluctuations to the actual rates, but at a consistently higher level.

Figures 5–9 compares the 2021 lung cancer incidence rate with the predicted values from various machine learning models. The predicted values generally align with the observed trend, except for linear regression (Figure 9), showing the predictions from machine learning models and DMD have a more robust and resilient performance in the face of noise and uncertainty. While linear regression captured a similar overall direction, its predictions deviated more significantly from the actual data. Overall, the agreement between the predicted and actual values was modest, as evidenced by the correlation coefficients in Table 1.

## 4 Discussion

This study used county-level lung cancer incidence rates to test DMD and other analysis methods, including linear regression, time series, and machine learning models. We found that DMD has a comparative prediction ability compared to other models while having low computational cost and skill set requirements. The results suggest that time series, machine learning methods, and linear regression models were able to capture the underlying trends in incidence rate data and generate reasonably accurate predictions for 2021. While the specific performance metrics may have differed slightly between the models, the overall trends and insights gleaned were largely similar.

In our study, DMD exhibited comparable prediction accuracy while requiring significantly less computational power and producing results faster than RF and SVM. By decomposing the data matrix into a set of dominant modes, DMD focuses on capturing the most essential information for representing the system’s dynamics. This reduces the computational complexity compared to machine learning models, which often involve complex non-linear optimization processes and require tuning numerous hyperparameters. These lower computational needs and faster processing time features of DMD make this method particularly appealing for users with limited resources or needing real-time data analysis. Additionally, due to its efficient processing format, DMD excels at identifying modes from time series data. This efficiency translates to lower training requirements than ML models while achieving comparable accuracy. In essence, DMD presents a trade-off between accuracy and computational demands, making it a choice for practical scenarios when high computational cost is not permitted.

Our comparison applying various ML models to lung cancer incidence prediction revealed some expected and unexpected differences. First, linear regression models often perform well with linear trend data. However, cancer incidence rate temporal trends are typically not strictly linear, posing a lower prediction ability. In our study, linear regression has the highest RMSE, showing the underlying temporal trends at the county level may be more complex than simple linear relationships. Second, time series typically shows the lowest RMSE as the model often detects the recurrent patterns in the temporal data, yet it performed the best in our study. Third, RF predicted cancer incidence rates better than SVM and GBM based on the RMSE scores. RF combines multiple decision trees, making it robust enough to handle complex relationships within the data. Fourth, like RF, GBM uses decision trees. However, GBM builds them sequentially, potentially leading to overfitting, especially with highly correlated features in cancer incidence data. The results indicate GBM and SVM may struggle compared to RF with complexities in data.

**Figure 10:**
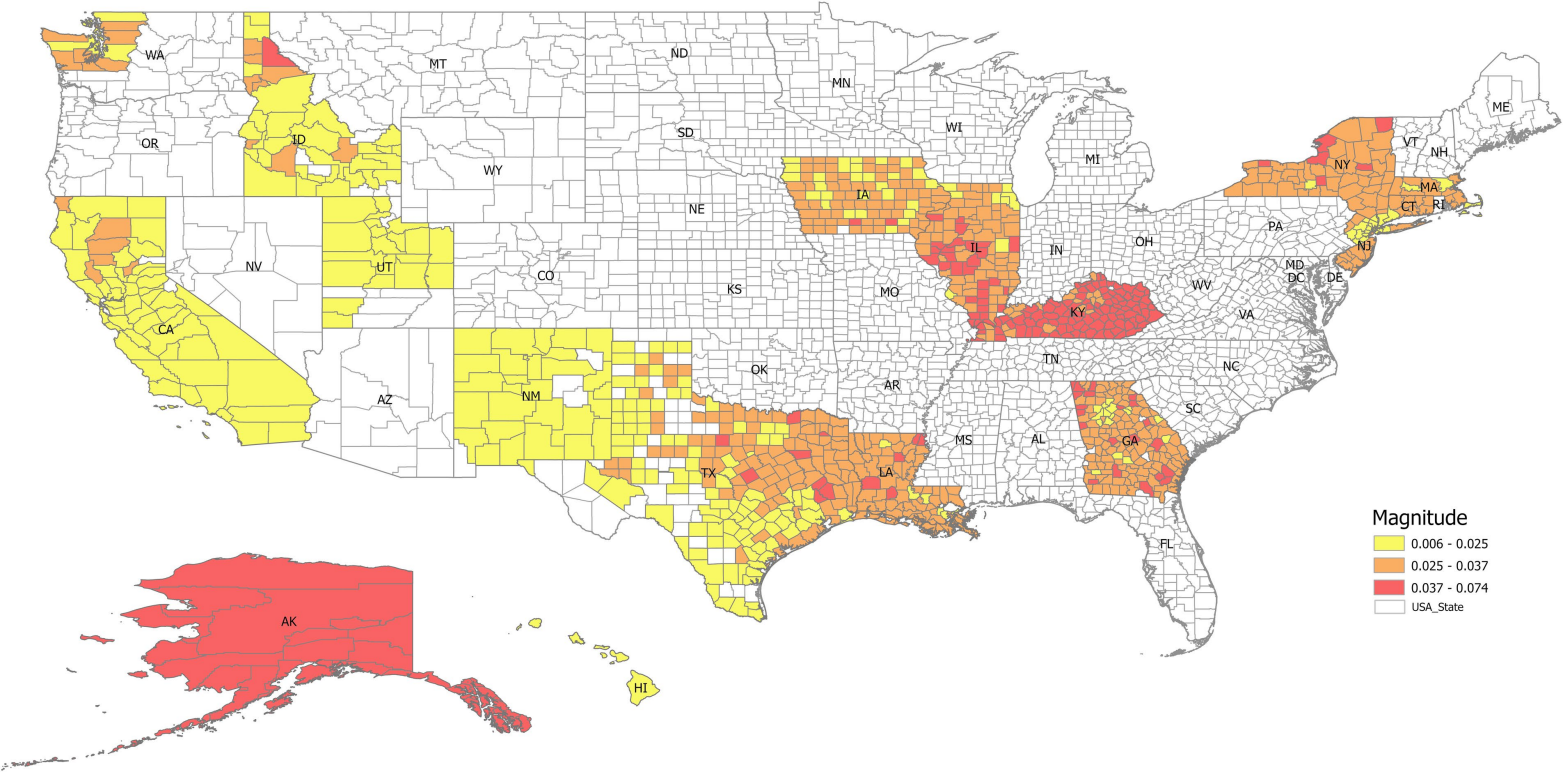
The magnitude of the dominant eigenvector (i.e., the steady state when the frequency is zero) mapped into different states. It indicates that nearly all the counties in Kentucky have a higher lung incidence rate, while California, New Mexico, Utah, and Idaho have lower trends.

An advantage of DMD is the interpretability of the extracted modes. For some ML models, the users cannot see the underlying logic, making interpretation challenging. With DMD, the dynamic modes directly correspond to specific frequencies and decay rates in the data. This allows users to understand the underlying geospatial-temporal patterns driving the observed trends and gain valuable insights into the system’s dynamics. The magnitude of the modes is a measure of the contribution to lung incidence rate by the local county under the associated eigenvalues. For example, the magnitude of the dominant eigenvector/mode (i.e., the frequency is zero) was mapped into different states, as shown in Figure 10. It indicates that nearly all the counties in Kentucky had a higher lung incidence rate, while California, New Mexico, Utah, and Idaho had lower trends. This interpretability is crucial in public health applications, where transparent and explainable results are essential for informing decision-making and intervention strategies (16–18). The extracted DMD modes come from the original data without further simulation or operation, offering a direct avenue to public health professionals and partners with limited data literacy. Public health practitioners often face the challenge of rapidly analyzing and responding to emerging public health threats like infectious disease outbreaks, biological emergencies, and natural disasters (17). These situations usually necessitate implementing time-sensitive interventions and resource allocation strategies based on real-time data analysis. In such scenarios, a ML model’s ease of use and interpretability become crucial factors. While neural networks and SVM offer powerful prediction capabilities, their complexity often requires significant expertise in model training, hyperparameter tuning, interpretation, and advanced computational ability (18), which can be a barrier for public health practitioners without extensive data science backgrounds (19–21).

With its inherent interpretability and relatively simpler implementation process, DMD emerges as a potential tool that public health professionals could readily adopt. The core algorithm of DMD relies on well-established linear algebra techniques that simplify the model implementation compared to ML models, which often require specialized coding skills and libraries. Additionally, visually analyzing the extracted dynamic modes associated with the data empowers users to understand the underlying patterns driving the observed trends. Moreover, DMD’s ability to handle complex and high-dimensional data makes it suitable for analyzing data sets encompassing various public health indicators, such as demographics, socioeconomic factors, and environmental data. By integrating these diverse data sources, DMD can contribute to developing more comprehensive and holistic public health models, ultimately aiding in identifying key risk factors and formulating targeted interventions. In contrast, RF and GBM are ensemble methods based on decision trees, adept at capturing complex, non-linear relationships but often sacrificing interpretability and requiring significant computational resources for training large ensembles. SVM seeks an optimal hyperplane for classification or regression, powerful for high-dimensional data but sensitive to kernel choice and parameter tuning, with interpretability linked to support vectors rather than system dynamics. TS focus specifically on temporal dependencies and trends using smoothing techniques, generally computationally efficient for univariate series but inherently lacking spatial awareness. DMD’s reliance on linear algebra often results in lower computational demands compared to the iterative optimization or ensemble building of RF, GBM, and SVM, particularly as data dimensionality increases, positioning it as a balanced method for dynamic system analysis where interpretability and efficiency are key considerations (Table 2).

**Table 2:** Comparison of dynamic mode decomposition with other models.

Feature	Dynamic Mode Decomposition (DMD)	Random Forest (RF)	Gradient Boosting Machine (GBM)	Support Vector Machine (SVM)	Time Series (TS)
Core principle	Linear dynamics approximation	Ensemble of decision trees	Sequential ensemble of trees	Optimal hyperplane separation	Exponential smoothing/ARIMA
Computational cost	Low	High (multiple trees)	High (sequential training)	Moderate-High (kernel/tuning)	Low (for univariate)
Spatial awareness	Yes (inherent in modes)	No (requires feature engineering)	No (requires feature engineering)	No (requires feature engineering)	No
Key strength	Dynamic insights, efficiency	Robust prediction, handles noise	High predictive power	Effective in high dimensions	Simplicity, trend capture

The potential applications of DMD extend beyond cancer incidence rate prediction and can benefit various public health domains. For instance, our previous study (4) demonstrated how DMD could be used to analyze and predict the spread of COVID-19 by identifying patterns in case data or mobility trends. Similarly, DMD could be applied to analyze and forecast trends in other health-related factors like obesity rates, mental health prevalence, or vaccine coverage, aiding in resource allocation and intervention planning. For example, DMD could be used to analyze data sets combining information on air pollution levels, socioeconomic factors like poverty rates, and asthma prevalence in a specific region. By identifying and interpreting the dominant modes in such a complex data set, DMD could reveal potential relationships between air pollution exposure, socioeconomic disadvantage, and asthma risk. Public health officials could use this information to prioritize air quality improvement efforts and target interventions.

Furthermore, combining DMD with geospatial analysis offers a unique advantage of visualizing high-risk areas. By overlaying the predicted incidence rates onto a map, we can readily identify geographic regions with high cancer rate prevalence. This visual representation can be a powerful tool for public health officials to address other public health crises, allowing them to target their interventions and resource allocation to identify high-risk populations and prepare for outbreaks.

### 4.1 Strengths and limitations

This study offers practical insights into the potential of machine learning techniques, particularly DMD, for analyzing and predicting public health trends. First, the utilization of real-world data on lung cancer incidence rates and readily available lung cancer incidence data adds context and strengthens the generalizability of the findings. Second, we compare the DMD with multiple ML models, using only lung cancer incidence rate data as the input, which provided a practical example and setting to test the DMD capacity. In fact, DMD has been applied to model infectious diseases such as flu (3), and COVID19 (4), which shows its capability in data driven modeling of high-dimensional spatial temporal analysis.

This study also has limitations. First, the study relies on a single data set of lung cancer incidence, limiting the generalizability of the findings to other populations or contexts. Additionally, the accuracy of the predictions generated by the machine learning models depended on the quality and completeness of the data used. Furthermore, as DMD uses linear modes to approximate nonlinear dynamic systems, its performance could be affected by highly nonlinear patterns. If the dynamic high-dimensional system is dominant with periodic or quasi-periodic behaviors, then DMD works well. However, if the system is strongly intermittent or sporadic, then other nonlinear models should be used. Further research involving larger and more diverse datasets across different geographic regions is warranted to validate the findings and solidify the applicability of DMD in public health settings.

## 5 Conclusion

In summary, this study examined the capacity of various data-driven models to predict lung cancer incidence rates, focusing particularly on DMD. We found DMD has a comparative analysis ability compared to more complex machine learning models. DMD can also offer both temporal and spatial insights into public health data, highlighting its potential as a convenient and effective analysis tool for a more comprehensive understanding of public health trends.

## Data Availability

The raw data supporting the conclusions of this article will be made available by the authors, without undue reservation.
